# Supplementary material for: Simulated Microgravity Exposure Induces Antioxidant Barrier Deregulation and Mitochondria Enlargement in TCam-2 Cell Spheroids
Source: Cells. 2023 Aug 19;12(16):2106. doi: 10.3390/cells12162106 (PMC10453291; doi:10.3390/cells12162106)
Supplement: Supplementary file 1 [file cells-12-02106-s001.zip › Berardini et al., Figure S1.pdf]

Microgravity exposure induces antioxidant barrier deregulation and mitochondria alterations in TCam2 cell spheroids

M. Berardini et al

Supplemntary material of Western blots:
In this section, the replicates of all immunoblots for each protein analyzed are reported. Where indicated, the same membrane was probed with different antibodies or stripped and re-probed with other antibodies. Representative blots shown in the paper are indicated with a red box.

A

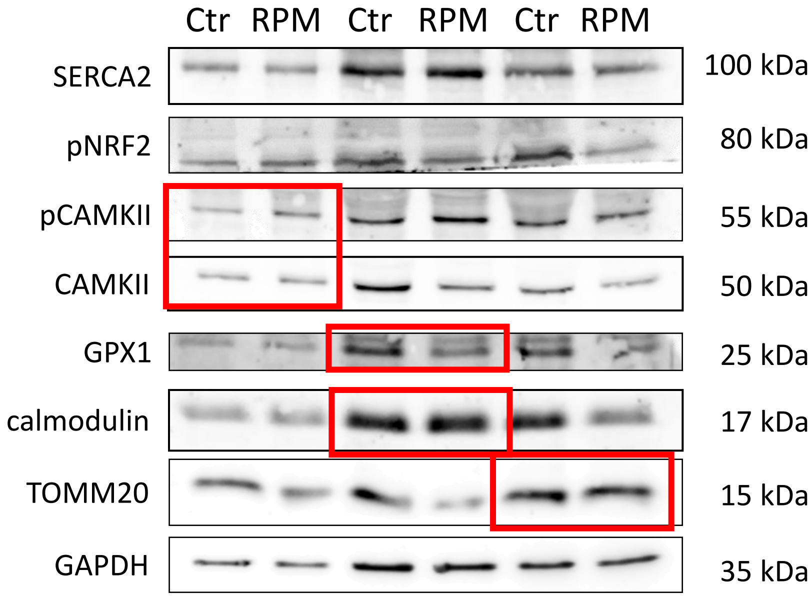

Merge of membrane A with markers

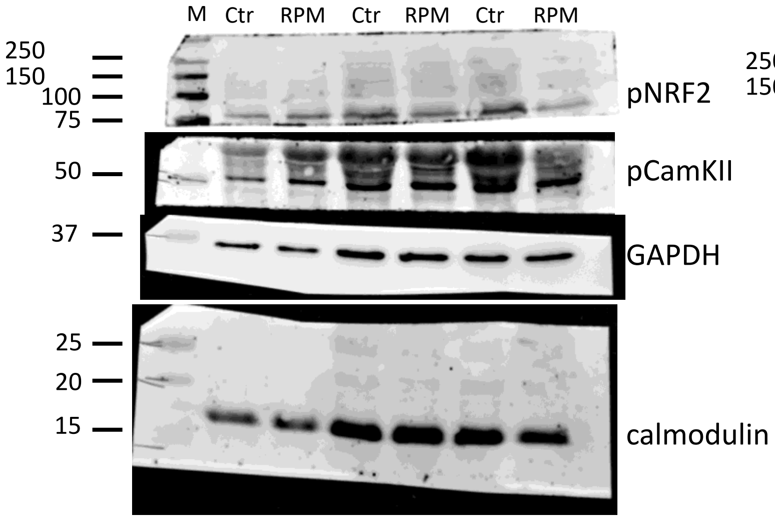

After stripping

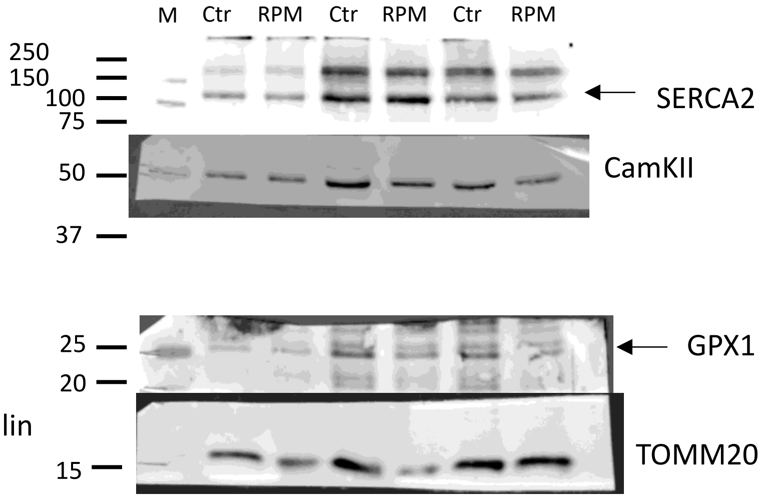

B

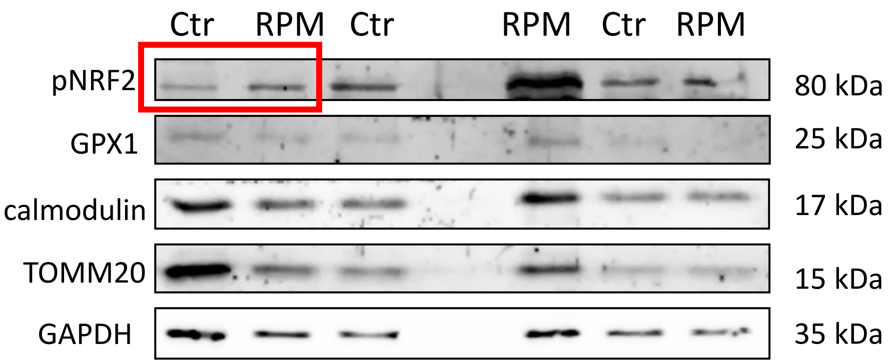

Merge of membrane B with markers

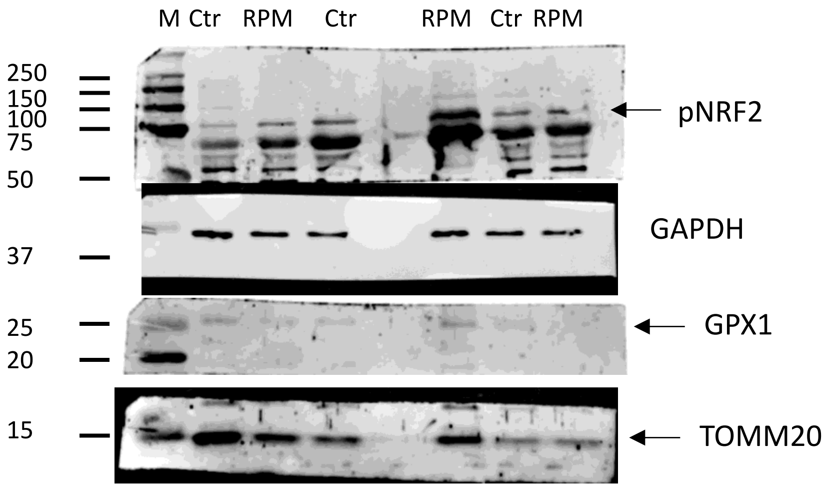

After stripping

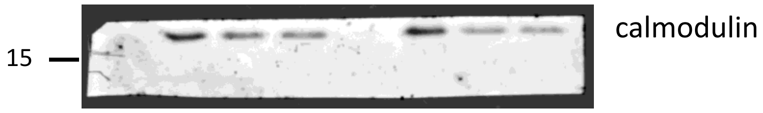

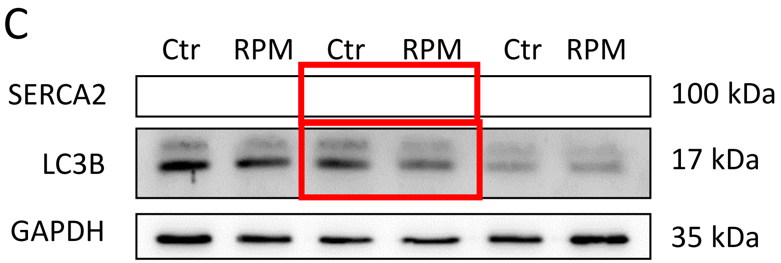

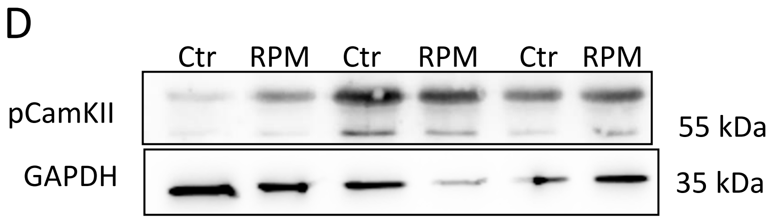

Merge of membrane C with markers

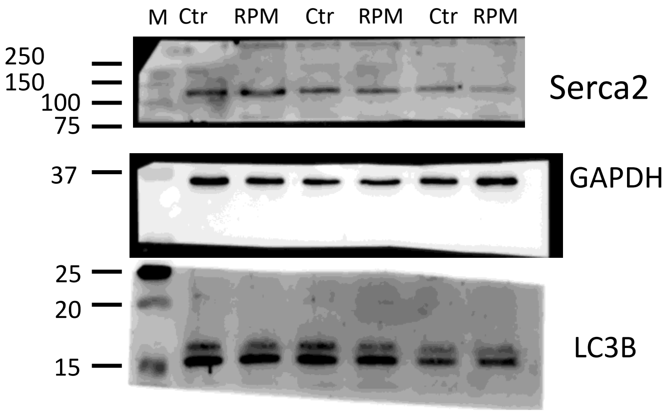

Merge of membrane D with markers

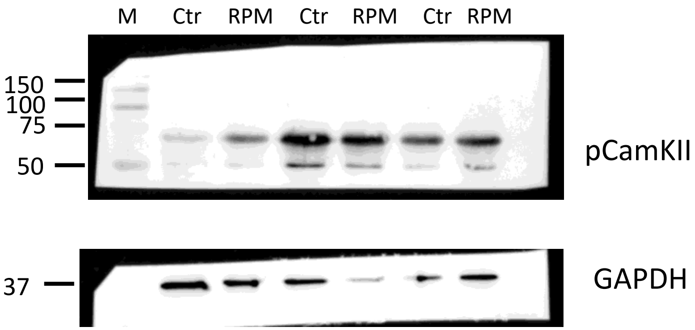

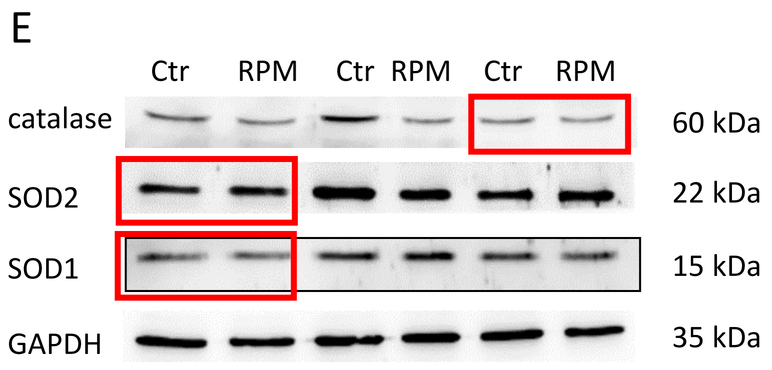

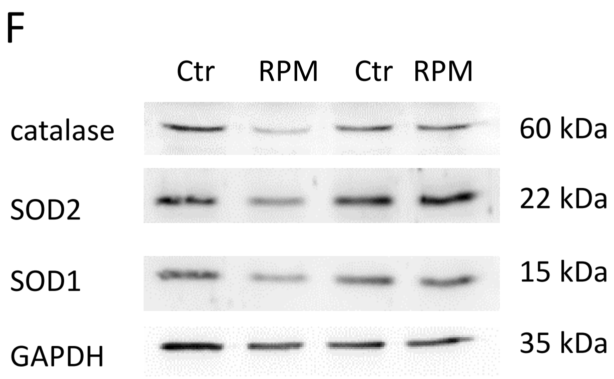

Merge of membrane E with markers

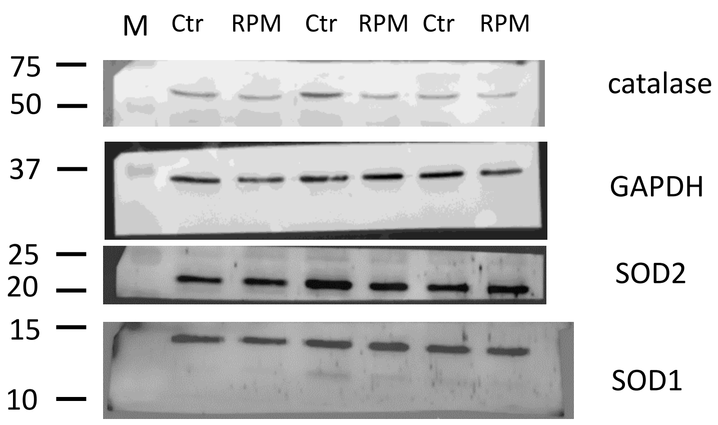

Merge of membrane F with markers

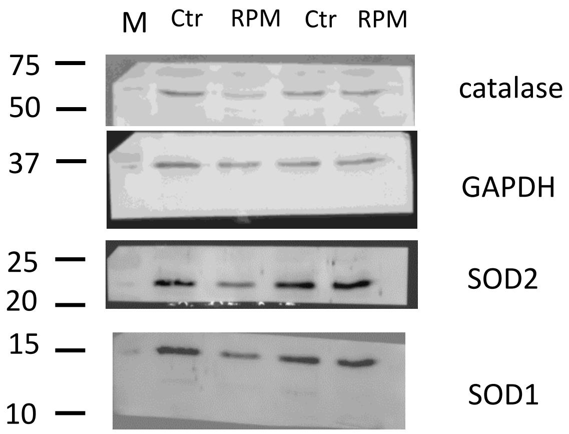

G

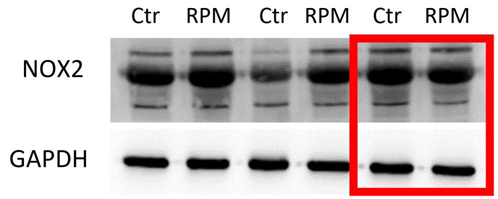

H

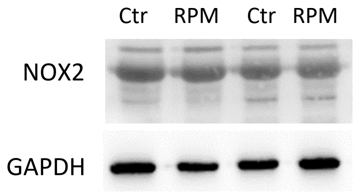

Merge of membrane G with markers

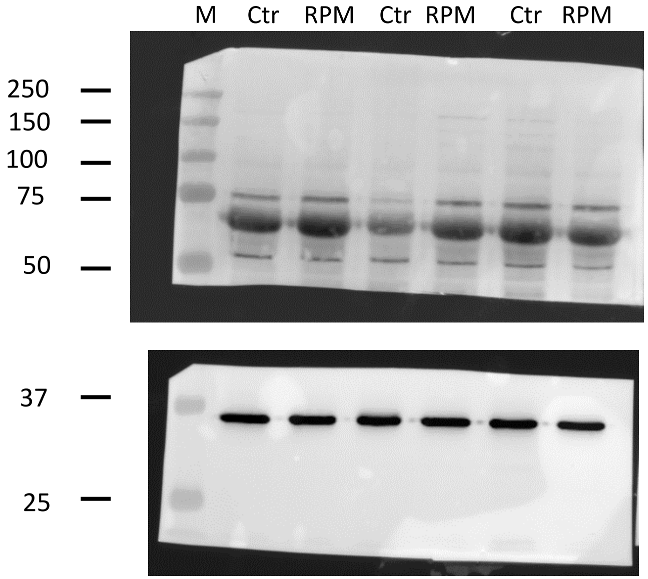

Merge of membrane H with markers

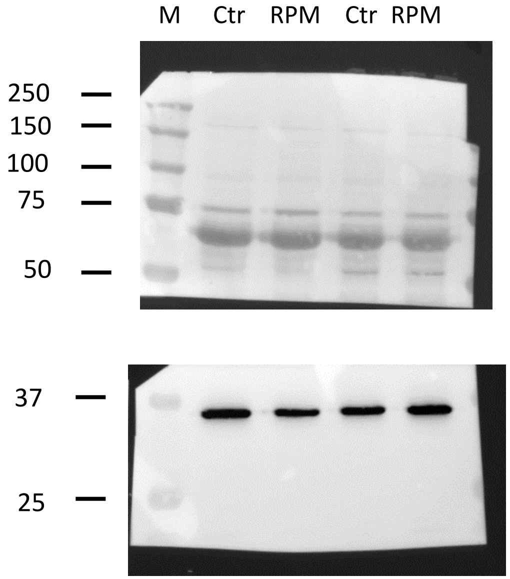

I

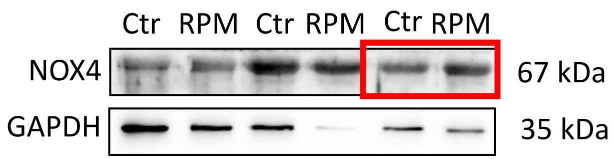

Merge of membrane I with markers

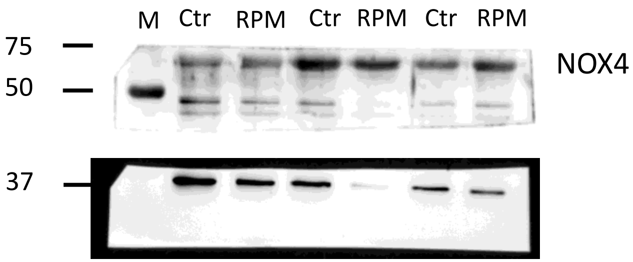

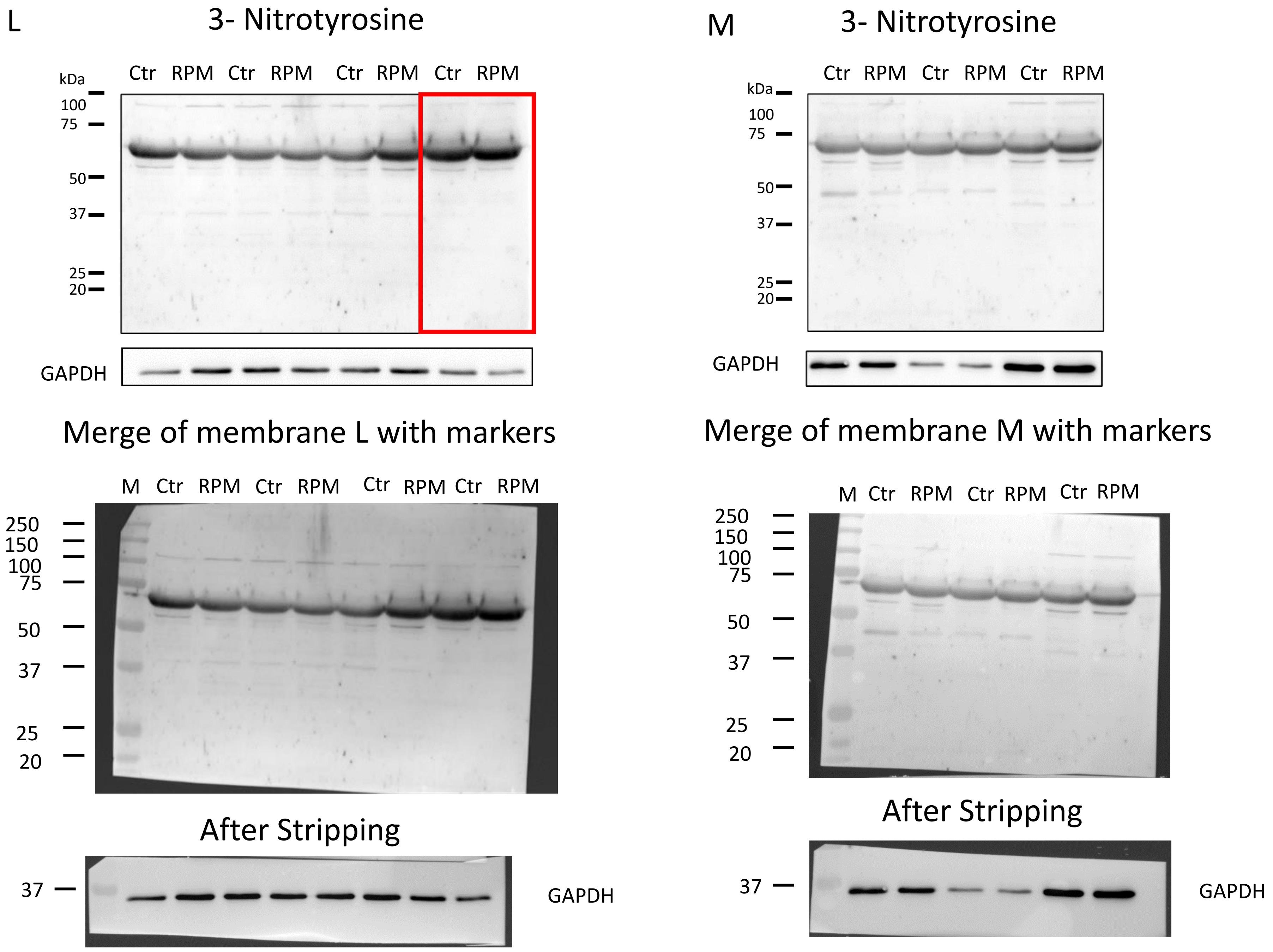
L
3- Nitrotyrosine
kDa
100
75
50
37
25
20
Ctr RPM Ctr RPM Ctr RPM Ctr RPM
GAPDH
Merge of membrane L with markers
M Ctr RPM Ctr RPM Ctr RPM Ctr RPM
250
150
100
75
50
37
25
20
After Stripping
37
GAPDH
M
3- Nitrotyrosine
kDa
100
75
50
37
25
20
Ctr RPM Ctr RPM Ctr RPM
GAPDH
Merge of membrane M with markers
M Ctr RPM Ctr RPM Ctr RPM
250
150
100
75
50
37
25
20
After Stripping
37
GAPDH

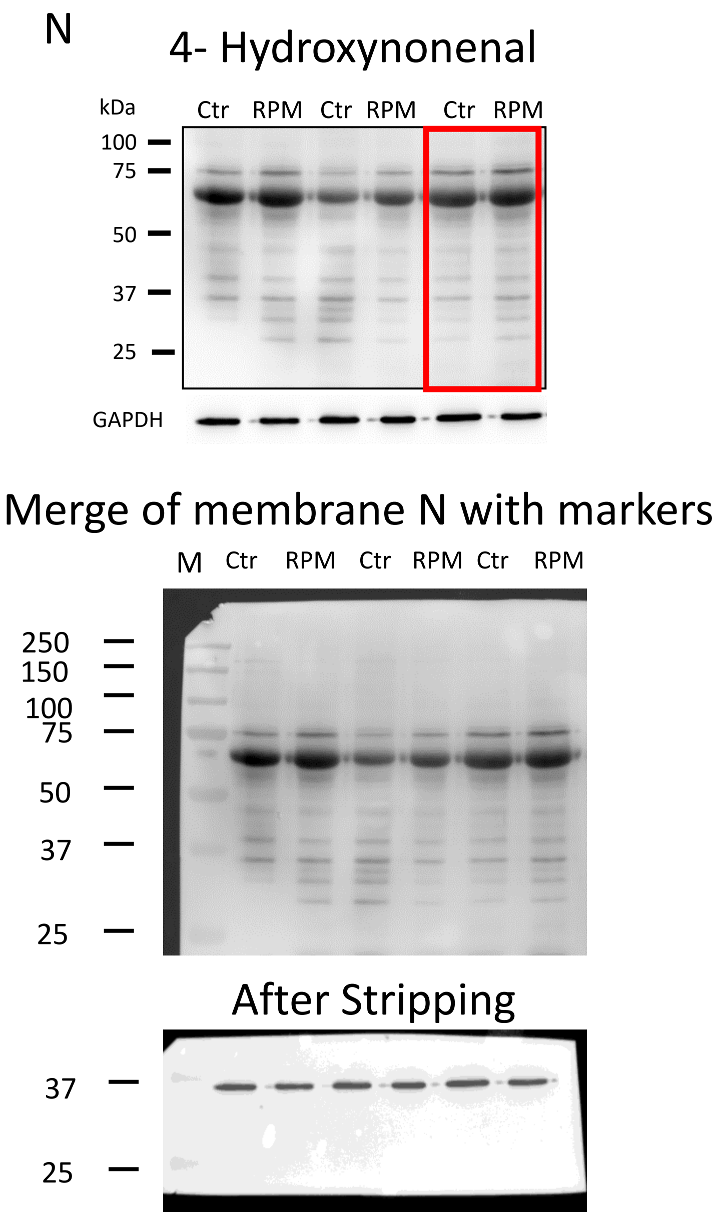
N
4- Hydroxynonenal
kDa
Ctr RPM Ctr RPM Ctr RPM
100
75
50
37
25
GAPDH
Merge of membrane N with markers
M Ctr RPM Ctr RPM Ctr RPM
250
150
100
75
50
37
25
After Stripping
37
25

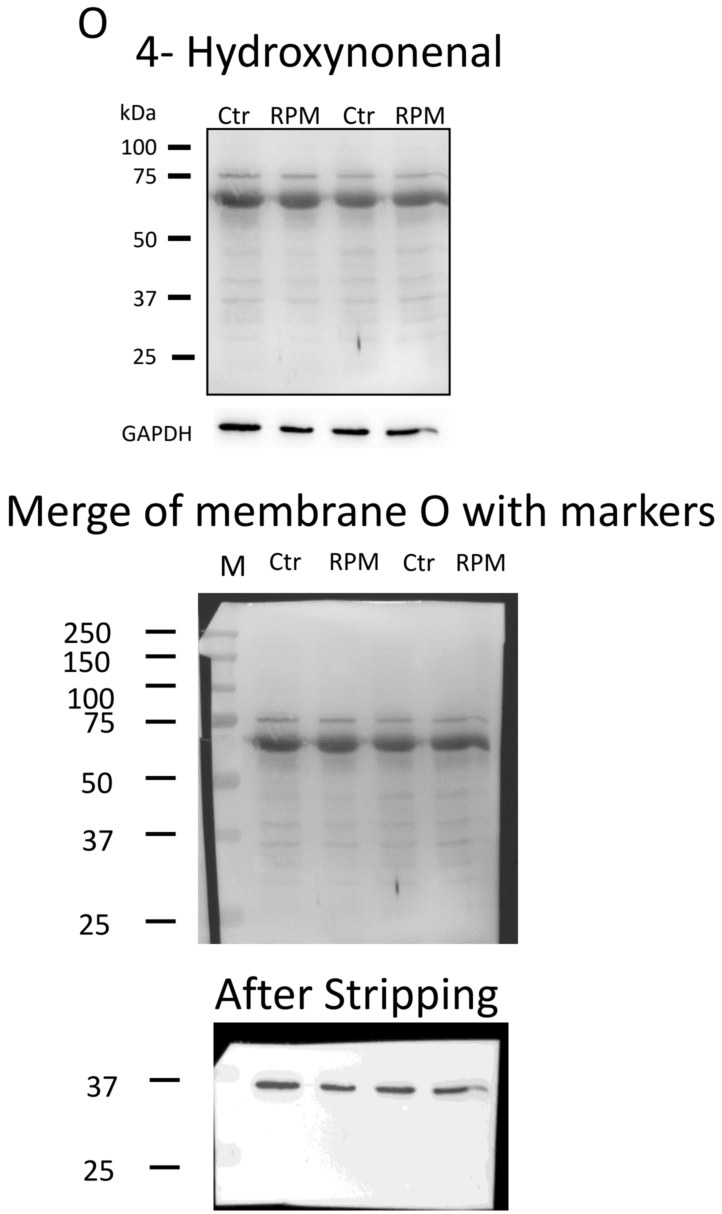
O
4- Hydroxynonenal
kDa
Ctr RPM Ctr RPM
100
75
50
37
25
GAPDH
Merge of membrane O with markers
M Ctr RPM Ctr RPM
250
150
100
75
50
37
25
After Stripping
37
25
