# Supplementary material for: Simulated Microgravity Exposure Induces Antioxidant Barrier Deregulation and Mitochondria Enlargement in TCam-2 Cell Spheroids
Source: Cells. 2023 Aug 19;12(16):2106. doi: 10.3390/cells12162106 (PMC10453291; doi:10.3390/cells12162106)
Supplement: Supplementary file 1 [file cells-12-02106-s001.zip › Berardini et al., Supplementary Table 1.pdf]

Target gene	Forward primer	Reverse primer
SOD1	5'-TGTGGGGAAGCATTAAAGGA-3'	5'-TGGATAGAGGATTAAAGTGAGGACC-3'
CAT	5'-CCACCTGAAGGATGCACAA-3'	5'-AGTCAGGGTGGACCTCAGTG-3'
GPX1	5'-CCGGGACTACACCCAGATGA-3'	5'-TCTTGGCGTTCTCCTGATGC-3'
HMOX1	5'-AACTTTCAGAAGGGCCAGGT-3'	5'- CTTGTTGCGCTCAATCTCCT-3'
XDH	5'-GGGGAACACCCAGGATCTC-3'	5'-GGAAGGTTGGTTTTGCACAG-3'
CYBA	5'-CCAGTGGTACTTTGGTGCCTA-3'	5'-GAGCCCTTCTTCCTCTTCCC-3'
CYBB	5'-CTGTCCTTCCTCAGGGGTTC-3'	5'-ATCATCCATGCCACCATTTT-3'
NCF1	5'-TCGACTTCTTCAAGGTGCG-3'	5'-TACTCTTGCCATCTTTGGGC-3'
NCF2	5'-GGGGCCAGGTGAAAAACTAC-3'	5'-TCATCTGGAAAGCCTTGGTC-3'
GUSB	5'-CGGTCGTGATGTGGTCTGT-3'	5'-GGTCCAAGGATTTGGTGTGA-3'

Table1
